# Perspectives on Stem Cell-Based Regenerative Medicine with a Particular Emphasis on Mesenchymal Stem Cell Therapy

**DOI:** 10.31662/jmaj.2021-0080

**Published:** 2021-12-24

**Authors:** Hisakazu Yamagishi, Kazuo Shigematsu

**Affiliations:** 1 Kyoto Prefectural University of Medicine, Kyoto, Japan; 2 Department of Neurology, Minami Kyoto Hospital, National Hospital Organization, Kyoto, Japan; 3 Nagitsuji Hospital, Kyoto, Japan

**Keywords:** Regenerative medicine, Mesenchymal stem cell, ALS, Alzheimer’s disease, Parkinson’s disease

## Abstract

Regenerative medicine is a medical treatment that aims to restore lost human body functions by regenerating missing or dysfunctional organs and tissues using stem cells, etc. There are three major types of stem cells used in regenerative medicine: induced pluripotent stem cells (iPS cells), embryonic stem cells (ES cells), and mesenchymal stem cells (MSCs). MSCs are expected to be widely applied to regenerative medicine because of their ability to differentiate into various types of cells, repair cells and tissues; anti-inflammatory effects; secretion of various growth factors; and resolution of abnormally accumulated protein amyloid. MSCs can be derived from bone marrow, dental pulp, and other sources, but adipose tissue-derived stem cells (ADSCs) may be superior in that they can be harvested with the least amount of invasion, and therefore, a sufficient amount of stem cells can be cultured relatively easily. When MSCs are administered systemically by intravenous infusion, they tend to accumulate at the site of disease, a property known as “homing,” which is extremely advantageous for clinical applications. In Japan, stem cell therapy can be performed only after the research or treatment plan has been reviewed and approved by the “Committee for Specific Approval of Regenerative Medicine” and submitted to the Ministry of Health, Labor and Welfare for approval in accordance with the “Act on Securing the Safety of Regenerative Medicine” and after approval by the ethics committee of the facility where the therapy is performed. In this review, the characteristics of MSCs, the actual status of their clinical application, and their future prospects are presented.

## Introduction

Regenerative medicine is a medical treatment proposed to regenerate body tissues that have been lost because of illness or accidents ^(1)^. The term “fundamental treatment” is often used, and it aims to fundamentally restore lost tissues and organs. Tissue engineering is “the technology to create replacements for organs and tissues that have lost their functions by combining life science and engineering.” Specifically, it is the idea of creating artificial organs and tissues by the following three elements: the patient’s “cells,” the “matrix” that provides a very important place (framework) for the cells to operate, and “bioactive substances,” which are nutritional components that have positive effects on our bodies. To artificially create organs and tissues, stem cells with strong differentiation ability are needed.

There are three main types of stem cells that are representative of those used in regenerative medicine. Induced pluripotent stem cells (iPS cells) ^(2), (3)^ and embryonic stem cells (ES cells) ^(4), (5)^ have this ability, and this is exactly why the creation of artificial organs and tissues and their clinical application have begun.

Another type of stem cell, mesenchymal stem cells (MSCs) ^(6)^, is also capable of differentiation, and a similar therapeutic approach may be established in the near future. Although MSCs have some advantages over the other two types of stem cells, such as not requiring genetic manipulation, no particular risk of cancer, and no ethical issues, they do not have a high differentiation potential and have not yet been applied to produce the desired tissue or organ. The main reason for the clinical application of MSCs is not the regeneration and transplantation of tissues and organs but the ability to repair damaged tissues and organs or the cells that make them up. MSC therapy consists of administration of undifferentiated stem cells, and iPS cells and ES cells are always differentiated before administration; this is the crucial difference between them, i.e., iPS cells or ES cells and MSCs.

## Stem Cell Types and the History of Their Discovery

There are three major types of stem cells used in regenerative medicine: iPS cells, ES cells, and MSCs. Because the first two have strong differentiation potential, they cannot be administered to the human body in their undifferentiated form, because if they were, they would undergo uncontrolled differentiation in the body and give rise to tumors such as teratomas, but they can be differentiated into specific cells and tissues and used in regenerative medicine as replacements for damaged cells and tissues.

iPS cells, like ES cells, have the potential to become any cell in the body. iPS cells are created by inserting a few genes (Oct3/4, Sox2, c-Myc, and Klf4) into adult skin or blood cells, whereas ES cells are obtained from a portion of an early fertilized egg, which requires the sacrifice of a fertilized egg. Most of them use fertilized eggs left over from in vitro fertilization, but the ethical problem of destroying fertilized eggs, the germ of life, is unavoidable.

## Clinical Application of iPS Cells

The clinical application of iPS cells has been progressing in Japan ^(7)^ probably because of their discovery in Japan and the Nobel Prize that was awarded to them. Because iPS cells are cells from another family, rejection can occur when they are transplanted. Two innovations are being attempted to reduce this rejection: one is to match human leukocyte antigen (HLA) types (iPS cells stock project for regenerative medicine ^(8)^), and the second is to use CRISPR-Cas9 genome editing technology to create iPS cells with less risk of immune rejection during transplantation in other families ^(9), (10)^. Current clinical applications include retinitis pigmentosa ^(11), (12)^, Parkinson’s disease ^(13), (14)^, and severe heart diseases such as dilated cardiomyopathy ^(15)^ and ischemic cardiomyopathy ^(16)^.

Takahashi et al. induced iPS cells from the skin cells of patients who had not responded adequately to existing therapies and then differentiated retinal pigment epithelial cells (RPE) to produce sheets ^(17)^. After the removal of the new blood vessel, the autologous iPS cell-derived retinal pigment epithelial cell sheets (iPS-RPE sheets) were transplanted into the retina. One year later, no tumor formation or rejection was observed, and no recurrence of neovascularization was seen. The patient maintained the visual acuity that he had before the transplant surgery. They are moving forward with a clinical study in which allogeneic iPS cells generated from human cells with a type of HLA that is less prone to immune rejection are transformed into RPE cells, and a suspension containing the cells is transplanted into the patient’s eye ^(13), (18)^.

When transplanting iPS cells into humans, it is especially important that the cells have differentiated into the target cells or, in the case of Parkinson’s disease, that dopaminergic progenitor cells have formed, that there are no undifferentiated cells left, and that there are no genetic abnormalities associated with cancer. In Parkinson’s disease, neurons in the substantia nigra of the midbrain are damaged, but iPS cells are transplanted into the striatum instead of the midbrain. The reason for this is that transplantation into the midbrain is technically risky, it is difficult for neurons differentiated from iPS cells to extend their neuronal processes into the striatum, and a certain effect is expected if dopamine is secreted in the striatum. Because the substantia nigra-striatal system is located bilaterally, the transplantation surgery should be performed bilaterally or, in other words, twice.

Sawa et al. conducted a study in which cardiomyocytes made from human iPS cells were processed into sheets and transplanted into pigs whose heart function was impaired because of ischemic cardiomyopathy, resulting in improved heart function ^(19)^. The first human subject was implanted with iPS cell-derived cardiomyocyte sheets in January 2020 ^(15)^. Because iPS cells can be differentiated into a variety of tissues, they have potential applications for a number of diseases. In this case, it is especially important that the cells are, first of all, fully differentiated, i.e., no undifferentiated cells remain; otherwise, they may differentiate against their intended purpose after transplantation, and tumor formation is a particular problem. Because iPS cells use genetic recombination technology, there should be no expression of abnormal genes after transplantation. Efforts to match HLA types are important to prevent rejection. To date, there has been no evidence of cancer, rejection, or abnormal gene expression in clinical applications. Of course, the application of iPS cells in nonclinical research, such as drug development, is one of the major applications of iPS cells, and in this case, “side effects” such as abnormal gene expression and canceration will not be as much of a problem as in transplantation.

## Mesenchymal Stem Cells

MSCs are adult stem cells that have the ability to differentiate into mesoderm-derived tissues such as bone, cartilage, blood vessels, and cardiomyocytes, as well as ectoderm-derived neurons and glial cells ^(20)^, and even endoderm-derived hepatocytes ^(21)^.

The International Society for Cellular Therapy uses the following criteria to define human MSCs: (1) adherence to plastic under standard culture conditions, (2) positive for the cell surface markers CD73, CD90, and CD105, and negative for CD11b or CD14, CD19 or CD79α, CD34, CD45, and HLA-DR, and (3) the ability to differentiate into osteoblasts, chondrocytes, and adipocytes ^(22)^.

MSCs have been found in a variety of body tissues, including bone marrow, muscle, skin, adipose tissue, umbilical cord, dental pulp, and periodontal tissue.

The first clinical application of MSCs was bone marrow transplantation of hematopoietic stem cells in 1957 by Thomas et al. ^(6)^. He was awarded the Nobel Prize in Physiology or Medicine in 1990, along with Joseph Murray, for establishing the treatment of blood diseases by bone marrow transplantation.

In 1966, Friedenstein et al. confirmed that allogeneic transplantation of bone marrow fragments and bone marrow suspension into mice resulted in the formation of reticular tissue and sometimes bone formation, following Denis’ report in 1958 that bone marrow transplanted subcutaneously stopped hematopoiesis, produced reticular tissue, and caused bone formation ^(23)^. It was in 1962 that John B. Gurdon, who was awarded the 2012 Nobel Prize along with Shinya Yamanaka for the creation of iPS cells ^(3)^, created a cloned frog by transplanting a somatic cell nucleus into a frog egg. ES cells were established in 1981 by Martin Evans, who cultured cells from the inner cell mass of a blastocyst, discovered pluripotent cells ^(4)^, and won the Nobel Prize in 2007. In 1999, Mark F. Pittenger et al. demonstrated the pluripotency of MSCs, i.e., that MSCs can differentiate into cartilage, bone, adipose cells, and muscle ^(24)^. De Bari et al. discovered synovium-derived MSCs ^(25)^, and Zuk et al. discovered adipose tissue-derived MSCs (ADSCs) ^(26)^, both in 2001. MSCs can be obtained from a number of tissues, including the umbilical cord, fetal appendages such as the placenta and amnion ^(27)^, and dental pulp ^(28)^.

Stem cells are present not only in childhood but also in adulthood, and throughout life, they function to replace damaged tissues. Among them, hematopoietic stem cells, which reside in bone marrow, were the first to be studied for more than half a century, and their clinical application has been actively pursued. The establishment of hematopoietic stem cell transplantation has opened up the possibility of transplantation therapy using any tissue stem cells. However, the difficulty of the isolation of stem cells from some tissues, for example brain and heart, makes it difficult to use them for therapy. Fortunately, though, MSCs have been found to have the ability to differentiate into not only mesodermal cells such as osteoblasts, adipocytes, myocytes, and cartilage cells but also endodermal cells such as visceral tissues and ectodermal cells such as neurons and are expected to be widely applied to “regenerative medicine.”

MSCs are cells that originally exist in the body. They are not genetically engineered nor are they artificially created cells, so it is unlikely that they have a higher chance of cancer. There are also no ethical issues in that they are not obtained by damaging embryonic cells. Although they are not pluripotent cells like iPS cells or ES cells, MSCs in the body play a major role in protecting and repairing tissues, calming inflammation, and recovering from diseases and injuries. Autologous stem cell administration, in which autologous MSCs are increased and returned to the patient, does not cause rejection.

## Characteristics of MSCs in Clinical Applications

- Ability to differentiate into several types of cells ^(24), (29)^

　When MSCs are isolated and cultured, they form a monolayer and show a certain phenotype, but when appropriate scaffolds and substances are added, such as β-mercaptoethanol, epidermal growth factor, nerve growth factor, and brain-derived growth factor in the case of neuronal differentiation, they differentiate into various lineages ^(30)^.

　1) Mesoderm-derived tissues such as bone, cartilage, blood vessels, adipocytes, and cardiomyocytes

　2) Ectoderm-derived neurons and glial cells ^(31)^

　3) Endoderm-derived hepatocytes ^(32)^

- Secretion of cytokines and trophic factors

　MSCs have been reported to secrete a variety of cytokines and growth factors.

　1) Cytokines, including IL-6, IL-8, IL-11, IL-12, IL-14, and IL-15 ^(33)^

　2) Platelet-derived growth factor (PDGF) ^(34)^

　3) Vascular endothelial growth factor (VEGF) ^(35)^

　4) Hepatocyte growth factor (HGF) ^(35), (36)^

　5) Brain-derived neurotrophic factor (BDNF) ^(37)^

　6) Nerve growth factor (NGF) ^(37)^

　7) Insulin-like growth factor-1 (IGF1) ^(38)^

　8) Stromal-derived growth factor-1 (SDF1) ^(38)^

　9) Granulocyte colony stimulating factor (GCSF) ^(33)^

　10) Granulocyte macrophage colony stimulating factor (GMCSF) ^(33)^.

- Anti-inflammatory effect ^(39), (40), (41)^

　In traumatic brain injury, intravenous administration of MSCs decreases microglia and proinflammatory cytokines at the extracellular site while increasing anti-inflammatory cytokines ^(42)^. IL-1β and TNF-α at the site of spinal cord injury were significantly decreased in the MSC group compared to the control group^(43)^.

- Protects and repairs neurons and promotes myelin sheath repair (oligodendrocyte protection) ^(34), (36), (37)^

　When rats with a 5-mm transection of the sciatic nerve were treated with MSCs, 70% of the treatment group had a percentage of the maximum diameter of axons crossing the nerve gap of 50% or more compared to 0% in the control group ^(44)^. Treatment of postnatal 9-day-old rats with hypoxic-ischemic brain injury, followed by MSCs 3 days later, improved neuronal and oligodendrocyte loss by 42% and 31%, respectively, 21 days after treatment. It also improved functional outcome and reduced lesion volume, indicating increased differentiation of recently divided cells into neurons and oligodendrocytes and reduced proliferating inflammatory cells ^(45)^.

- Revascularization

　MSCs promote proliferation of vascular endothelial cells and inhibits apoptosis ^(35)^. One week after myocardial infarction, autologous MSCs were administered intramyocardially to the infarcted area, and two months later, the expression level of VEGF was significantly increased, vascular density and regional blood flow in the infarcted area were increased, apoptosis of hypertrophic cardiomyocytes was decreased, and contractility of the left ventricle was markedly improved ^(46)^.

- Nephroprotective effect

　Inhibition of infiltrating macrophages and induction of immunomodulatory macrophages at the site of nephritis ^(47)^.

- Degradation of abnormal proteins

　MSCs have neprilysin activity, an enzyme that degrades amyloid-β, an insoluble protein deposited in the brain in Alzheimer’s disease ^(48)^. Repeated intravenous administration of MSCs has improved cutaneous amyloidosis ^(49)^.

　Abilities that provide evidence that MSCs are effective for clinical applications are summarized in Table 1.

**Table 1.** Abilities That Provide Evidence That MSCs Are Effective for Clinical Applications Are the Following.

1	to differentiate into multiple cell lineages
2	to secrete a number of cytokines and trophic factors
3	to regulate inflammation
4	to protect and promote the repair of neurons and myelin sheaths
5	to improve blood circulation
6	to protect the kidney
7	to degrade abnormal proteins, including amyloid β.

Furthermore, MSCs are being studied for use in the field of tissue engineering. The aim of tissue engineering is to create three-dimensional artificial organs and tissues by appropriately combining (1) cells, (2) platforms, and (3) nutrition ^(31)^.

The treatment of chondral injuries with cartilage cell sheets using cells differentiated from MSCs is already underway ^(50), (51), (52)^.

## Advantages of Adipose Tissue-Derived Stem Cells

ADSCs can be harvested from subcutaneous fat with minimal invasion under local anesthesia. It has been reported that about 500 times more MSCs can be obtained from adipose tissue than from the bone marrow tissue of the same volume ^(53)^ and that adipose tissue produces more growth factors (regeneration-promoting factors) such as HGF and VEGF that contribute to organ repair than bone marrow-derived tissue ^(35)^. ADSCs have a higher immunomodulatory capacity than those derived from bone marrow ^(54)^.

## Clinical Applications of MSCs

MSCs can be harvested from bone marrow or subcutaneous adipose tissue, and their use in regenerative medicine is increasing as they are easier to apply clinically than ES cells or iPS cells due to fewer safety and ethical issues. In Japan, nonautologous bone marrow-derived MSCs (Temucel^Ⓡ^ HS Injection, JCR Pharma K.K.) were approved for the treatment of acute graft-versus-host disease (GVHD) after hematopoietic stem cell transplantation in 2015, and they are administered as an insurance-approved treatment for patients with acute GVHD. In addition, in 2018, it was approved as a treatment for acute GVHD. Furthermore, in the same year, autologous bone marrow-derived MSCs (Stemilac^Ⓡ^ Injection, Ni Co., Ltd.) were approved as a regenerative medicine product for traumatic spinal cord injury, and treatment under health insurance became possible.

In Japan, the “Act on Securing the Safety of Regenerative Medicine” (https://www.mhlw.go.jp/english/policy/health-medical/medical-care/dl/150407-01.pdf) was enacted in November 2014 along with the “Act on Securing the Quality, Efficacy and Safety of Pharmaceuticals and Medical Devices, etc.” with the aim of promoting the safety and development of regenerative medicine. The Ministerial Ordinance for Partial Revision of the Ordinance for Enforcement of the Act on Securing the Safety of Regenerative Medicine and the Ordinance for Enforcement of the Clinical Research Act (Ordinance of the Ministry of Health, Labor and Welfare No. 14 of 2021) came into effect on February 1, 2021. These laws allow regenerative medicine using stem cells to be performed even if it is not covered by insurance (the principle of medical treatment in Japan is to be covered by insurance, and all citizens are insured) as long as it is discussed and approved by the Committee for Approval of Specific Regenerative Medicine, etc., and then approved by the Ministry of Health, Labor and Welfare. This therapeutic research requires the creation of a protocol, preparation of explanatory documents for the patients to be treated, explanation to the patients, and obtaining consent from the patients, and the results are treated in the same way as a clinical trial in determining whether to apply for insurance coverage. All of the treatments and studies approved in this way are posted on the Ministry of Health, Labor and Welfare’s website for anyone to view (https://www.mhlw.go.jp/stf/seisakunitsuite/bunya/0000186471.html). The “Regenerative Medicine Consultation Room” website summarizes these studies and treatments by target organ (http://www.rm-promot.com/).

Hundreds of therapeutic studies are currently underway, supported by the legal system described above. Even for spinal cord injuries, which are difficult to treat, improvement in motor and sensory dysfunction was reported with the administration of MSCs ^(55), (56)^. Furthermore, for neurodegenerative diseases such as Alzheimer’s disease ^(49)^, ALS, Parkinson’s disease, and intractable pulmonary diseases such as COPD and pulmonary fibrosis, for which there is no treatment to inhibit disease progression, therapeutic studies are being conducted using repeated intravenous administration of MSCs, especially autologous ADSCs, and the therapeutic effects are expected to be verified. As of August 2021, the patient with ALS reported in the case report, who was presented at the 40th World Congress of the International College of Surgeons (ICS) in 2016 (October 23-26) (Program & Abstracts p. 139 Hisakazu Yamagishi) is alive and well without a respirator or gastrostomy, although he is walking with a cane ^(57)^. Deposition of abnormal proteins in the brain has been found in many neurodegenerative diseases. The ability of MSCs to remove these abnormal proteins, together with their ability to repair neurons and myelin, may offer hope for the treatment of a number of neurodegenerative diseases that would otherwise be progressive and untreatable ^(58)^. Clinical research on MSCs has been very active, and their effects have been reported successively. Repeated infusion of ADSCs for Parkinson’s disease has been reported to improve MDS-UPDRS, although it is an open-label pilot study ^(59)^. Double-blind randomized controlled trials, often costly and sometimes ethically challenging, are the golden standard for determining clinical efficacy. If MSCs are given for progressive disease and symptoms improve or stop progressing, the effect may be considered in comparison to the best that could be achieved without the treatment. Even so, further validation is needed and well worthwhile.

## Methods of Administration of MSCs

MSCs can be administered systemically without transplantation to the target organ for two reasons: they can be administered in an undifferentiated state, which is not possible with iPS cells or ES cells, and they have a unique feature that allows systemically administered MSCs to accumulate in the lesion, known as homing. MSC homing is defined as the arrest of MSCs within the vasculature of a tissue, followed by transmigration across the endothelium ^(60)^. When MSCs were administered intravenously to rats with spinal cord injury, administered cells were found in the injured area as early as 6 h after administration and were confirmed until at least 28 days later ^(61)^. Similarly, when MSCs differentiated into oligodendrocyte precursor cells were administered intravenously to spinal cord-injured rats, about 30% of the administered cells accumulated in the injured area of the spinal cord after 4 weeks of treatment. At this time, 8% of the cells had migrated to the brain ^(62)^.

At this time, 8% had migrated to the brain, 12% to the kidneys, 7% to the liver, and 3% to the lungs ^(62)^.

## Safety of MSC Administration

Although MSCs are generally considered safe, with no tumors, including teratoma, and little rejection, especially for autologous stem cells, they express tissue factor (TF), a potent activator of coagulation ^(63)^. MSCs promote a prothrombotic state after exposure to blood, in which the mechanisms are attributed to the so-called instant blood-mediated inflammatory reaction (IBMIR) ^(64)^. The tissue factor expression and procoagulant activity of MSCs vary depending on the tissue origin, more so in adipose than in bone marrow, and on culture time. There is a risk of embolization with intravenous MSCs ^(65)^. Because intravenously administered MSCs go from the vena cava through the right atrium and right ventricle to the lungs first, it is necessary to be careful of pulmonary embolization. The risk of pulmonary embolism is associated with the number of cells administered and the rate of administration ^(66)^. MSCs, especially autologous MSCs, are considered safe, but because they have only been used for treatment for a rather limited time, there may be unknown side effects. Of course, some invasion, however small, will be added at the time of collection. Because bone marrow harvesting is necessary for bone marrow-derived MSCs and subcutaneous adipose tissue harvesting is necessary for ADSCs, attention must be paid to the safety of such procedures. Although not limited to stem cells, there should be no infection in the culture, and components of xenogeneic animal origin should be avoided when administered to humans.

## Autologous MSC vs. Allogeneic MSC

The advantage of administering allogeneic MSCs is that stem cells can be harvested and cultured beforehand, and then administered promptly when treatment is needed. This is especially important in the treatment of acute diseases, such as Covid-19, that cannot wait for the stem cell culture period.

On the other hand, with regard to the immunosuppressed state after allogeneic MSC treatment, an increased frequency of cytomegalovirus antigenemia ^(67)^ and an increase in pneumonia-related deaths have been reported ^(68)^. After the administration of allogeneic MSCs, it is necessary to take sufficient countermeasures against infectious diseases such as invasive fungal infections. There have been reports of the development of EB virus-associated posttransplant lymphoproliferative disease as well as EB viremia ^(69)^.

Because autologous MSCs have a wide variety of effects and are free from ethical problems and rejection, they will continue to develop as an attractive therapeutic method, and it will become clearer what method of administration is best, the best number of cells to be administered, the number of times to be administered, and the duration of administration, such as how long repeated administration should be continued. If it becomes clear which diseases can be improved by which mechanism of action, the range of indications will expand, and it may lead to the investigation of the causes of diseases for which the causes are still unknown.

## Conclusion

The potential of MSCs in regenerative medicine is great, which diseases they work on and to what extent, yet the mechanisms of their effects and specific methods of administration will be clarified in the future. In addition to their ability to differentiate into specific cells, MSCs may be able to secrete various growth factors and cytokines, remove abnormal protein deposits from tissues, and accumulate at the site of disease, a property known as homing. It is believed that MSC therapy will provide a ray of hope for the treatment of some intractable diseases for which there is currently no fundamental treatment and will hopefully reach patients suffering from such diseases.

## Article Information

### Conflicts of Interest

None

### Author Contributions

Both authors contributed equally to this article; H.Y. initiated regenerative medicine and K.S. wrote the manuscript. Both authors have read and approved the final manuscript.
